# Herpes Zoster

**Published:** 1890-03-15

**Authors:** 


### The Practitioner's Mirror and Retrospect.

[*The Editor will be glad to receive offers of co-operation and contributions from members of the profession. There is no doubt that much valuable material full of interest and instruction is at present lost to science. This is largely so in the case of* (1) *the younger and less-known members of the hospital staff* (2) *those connected with cottage hospitals, and* (3) *the great body of medical practitioners not attached to any institution. The co-operation of all is cordially invited to enable the Editor to make* The Hospital *of the utmost possible use and value to the profession. Specialists willing to review books on their special subjects are invited to communicate this fact at once. All letters should be addressed to* The Editor, The Lodge, Porchester Square, London, W.]

### MEDICINE.

### HERPES ZOSTER.

A case of recurrent shingles is reported in the *Illustrated Medical News* by Mr. Percy E. Wallis, surgeon to the East Grinstead Cottage Hospital. The patient, a female, had been subject to occasional eczema of the legs, associated with varicose veins, but with this exception enjoyed good health. In October, 1886, she had her first attack of herpes, consisting of a copious eruption of vesicles, accompanied by tingling and burning. In November, 1887, there was another severe attack in the course of the sixth, seventh, and eighth intercostal nerves, and as this eruption disappeared, a fresh one presented on the outside of both thighs and legs. In November, 1888, the third attack occurred, the parts affected being the left intercostal and both posterior scapular regions. Mr. Wallis remarks that the occurrence of repeated attacks of herpes of the trunk is unusual. But the family history of the patient showed a powerful hereditary predisposition to disease, her father, mother, four brothers, and two sisters having died of phthisis. Now, we do not think the recurrence of herpes is so uncommon as Mr. Wallis imagines. We can recall several instances of the kind. *Herpes labialis* and *herpes præputialis* frequently recur, and there is no valid reason why *herpes zoster* should not recur, especially in persons of the gouty or rheumatic diathesis; and more particularly when such persons are "below par" from other causes.

